# Developing Mixed Reality Educational Applications: The Virtual Touch Toolkit

**DOI:** 10.3390/s150921760

**Published:** 2015-08-31

**Authors:** Juan Mateu, María José Lasala, Xavier Alamán

**Affiliations:** 1 Universidad Autónoma de Madrid, Madrid 28049, Spain; E-Mail: juan.mateu@estudiante.uam.es; 2 IES Ernest Lluch, Cunit 43881, Tarragona, Spain; E-Mail: mlasala3@xtec.cat

**Keywords:** virtual worlds, tangible user interfaces, mixed reality, education

## Abstract

In this paper, we present Virtual Touch, a toolkit that allows the development of educational activities through a mixed reality environment such that, using various tangible elements, the interconnection of a virtual world with the real world is enabled. The main goal of Virtual Touch is to facilitate the installation, configuration and programming of different types of technologies, abstracting the creator of educational applications from the technical details involving the use of tangible interfaces and virtual worlds. Therefore, it is specially designed to enable teachers to themselves create educational activities for their students in a simple way, taking into account that teachers generally lack advanced knowledge in computer programming and electronics. The toolkit has been used to develop various educational applications that have been tested in two secondary education high schools in Spain.

## 1. Introduction: State of the Art

The educational world has been profoundly changed due to the emergence of the Information and Communication Technologies. Various computer technologies, from multimedia to the Internet, have had a deep impact on many aspects of education. Some of them, such as the creation of content on the Web or the use of Learning Management Systems (e.g., Moodle) have been already adopted as a standard practice worldwide.

Two frontier technologies that are currently being introduced in the educational area are Virtual Worlds and Tangible Interfaces. Both of them have been successfully applied in many prototypes and test cases, as will be described below. However, much less experience has been obtained yet in using these two technologies together, creating a new user experience that can be named as “mixed reality”.

This section will analyze the state of the art in both technologies as applied to the educational world, as well as review some of the initial experiences realized in combining both in order to create “mixed reality” systems.

### 1.1. Virtual Worlds

Different studies show the benefits of using virtual worlds in educational settings, where it is possible to develop a variety of innovative teaching and learning activities. Dickey [1] indicates that virtual worlds allow learning experiences under the constructivist pedagogical approach [2], by means of the interaction between avatars and the virtual environment. Dede [3] discusses the advantages of developing activities that have no undesired repercussions or consequences in the real world, while knowledge is obtained by “learning by doing” in a secure way, with a sense of immersion in an environment similar to reality.

The constructivist approach of Jonassen [4] states that learning, in order to be meaningful, has to be based in the active participation of the student. This can be achieved, for example, by manipulating physical artifacts with the objective of building a product, or by taking decisions that have some kind of consequence in the physical world. 

Virtual Touch, being a mixed reality system, allows building 3D collaborative spaces in a virtual world where students interact with each other and can perform an active role in the resolution of a given problem. Workgroups can be defined where students create and share the knowledge. Furthermore, the tangible interfaces provided by Virtual Touch allow the manipulation of physical artifacts that will have effects in the virtual world. In this way the student will be able to explore, experiment and interact in the virtual world using physical manipulation, thus being able to watch the immediate effects of her actions, in a constructivist learning environment.

Babu *et al.* [5] show that the interaction using avatars allows improving the skills of verbal and nonverbal communication. In addition, according to Peterson [6] the interaction in the virtual world through avatars facilitates communication and collaboration, allowing a sense of “telepresence” and a greater commitment from students. Furthermore, Annetta and Holmes [7] found that students with a sense of “telepresence” have a greater degree of satisfaction with the course.

Virtual worlds, depending on their use, can be classified mainly into three categories [8]: communicative spaces, simulation spaces and experimental spaces. As a communicative space, the focus is in the communication that can be achieved through the avatars, both in a verbal way (using text chat and voice chat) and in a non-verbal way, through the appearance of the avatar, as well as through the avatar postures and gestures. As a simulation space, the focus is in the virtual representation of places such as a university, a library, a theater, *etc*., where avatars can meet and interact with each other. As an experimental space, the focus is in the paradigm of “learning by doing”, interacting with 3D virtual objects or exploring the virtual world.

The educational activities that can be carried out in a virtual world are quite diverse [9]: exploration (simulation of a library, school, church); practicing with theoretical concepts through interaction (with other avatars, with objects in simulated locations); carrying out activities that are difficult to perform in the real world either for their difficult implementation or dangerousness (e.g., simulation of a medical intervention, handling chemicals); “role playing” activities, assigning different roles to each student; and finally, playing serious games, in which the student learns in a playful way.

**Table 1:** Educational virtual worlds.

Cat. *	Area	Project	Target	Virtual World	Website or Reference	Timing
**C**	Foreign Language learning	Kamimo Project	Higher education	Second Life	[10]	2007–2008
C		AVALON Project	Language teachers/learners	Second Life	[11]	2009–2011
C		AVATAR Project	Secondary school teachers	Second Life	[12]	2009–2011
C		NIFLAR	Secondary education	Second Life, OpenSim	[13]	2009–2011
C		TILA Project	Secondary education	OpenSim	[14]	2013–2015
C		CAMELOT Project	Language educators	Second Life	[15]	2013–2015
S	Art, Creativity	ST.ART	Secondary school	OpenSim	[16]	2009–2011
E	Mathematics and Geometry	TALETE Project	Secondary school	Unity	[17]	2011–2013
E	Programming	V-LEAF	Secondary school	OpenSim	[18]	2011
E		OpenSim & Scratch40S	Secondary school	OpenSim	[19]	2014
S,E	Science, biology, chemistry, history	The River City	Middle school	Active Worlds	[20]	2002
S,E		EcoMUVE	Middle school	Unity	[21]	2010
E	Collaborative, social skills	The Vertex Project	Primary school (K-12)	Active Worlds	[22]	2000–2002
E		Euroland	Primary and secondary	Active Worlds	[23]	1999–2000

* Categories: C: communicative space; S: simulation space; E: experimental space.

One area where virtual worlds have been used successfully is inclusive education. For example, the “Accessibility in Virtual Worlds” project is aimed at blind students whose positions are indicated by sounds, thus allowing navigation and interaction with peers (both blind and sighted) [24]. Another example that uses virtual worlds for inclusion is Brigadoon. It is an island in Second Life which serves as a therapy for people with Asperger syndrome (autism), facilitating their social interaction by using avatars in a controlled environment [25].

Table 1 shows a selection of some representative educational projects that use virtual worlds. The selection has been made among projects funded by public educational administrations, trying to cover several different areas of application:

- Several educative levels: primary school, secondary school and university.
- Several subject areas: mathematics, programming, *etc.*
- Several methodologies: Machinima, problems solving, *etc.*
- Several virtual world environments: Second Life, Open Sim, Active Worlds.

Notably, 40% of the reviewed projects are aimed specifically at foreign language teaching. Projects for teaching science and biology cover an additional 20%, and projects for teaching programming a 15%. The rest are multidisciplinary projects that work various skills (social skills, communication skills, creative skills, *etc.*) Although this mix cannot be extended to all the existing projects worldwide, probably the tendencies are representative. 

In Table 1 it can also be appreciated that the most used technology for virtual worlds among the selected projects is Second Life, or its open source version OpenSim (33% each, together more than 65% of the reviewed systems). The other two technologies employed in these projects are much less used: Active Worlds (20%) and Unity (15%). Again, the tendencies are representative: Second Life and Open Sim are the most used environments for virtual worlds.

Another interesting tendency that can be found in Table 1 is that 85% of the reviewed projects were conducted in Europe, compared with 12% in the USA. The European Commission (within the Lifelong Learning Program) funded 60% of them, which is evidence about the support of Europe to this technology.

### 1.2. Tangible Interfaces in Education

Fitzmaurice [26] introduced the term “graspable user interface” as a new paradigm that allowed direct control of virtual objects by manipulating physical devices, allowing a more direct and natural interaction. Ishii [27] proposed the term “tangible user interface” as an evolution of the concept, allowing the incorporation of digital information into everyday physical objects in a non-intrusive and ubiquitous way. Various educational applications have attempted to exploit this paradigm.

The main advantages that tangible interfaces provide for education applications are:

- Better understanding of complex structures through direct manipulation.
- Learning is more effective and natural.
- Tangible interfaces facilitate active participation and reflection.
- Accessible Learning is enabled (students with disabilities and students with special educational needs).
- Promotes collaborative learning through the development of collaborative spaces where the interaction among students is enforced.
- The results of the activities are more immediate, visible and palpable.
- Physical restrictions of the objects can prevent or reduce errors (e.g., programming blocks that do not fit because they can not go together).
- The activities with tangible interfaces strengthen motor skills.

Papert [28], under his constructivist approach, asserts that children learn more effectively building their own knowledge through physical manipulation of the environment.

These advantages can be found in the tangible interfaces educational projects that are reviewed below.

Webkit [29] is an application for improving children rhetorical skills, by means of using RFID cards containing different statements about a particular topic being discussed. Using these cards (arguments) children learn to argue about complex issues.

“Ely the Explorer” [30] uses a set of tangible elements to encourage collaborative and social activities among students, exploring different cultures through some fictional characters called Ely.

KidPad [31] is a collaborative drawing tool aimed at children between 5 and 7 years. KidPad, using a “magic carpet”, allows collaborative storytelling in a quite intuitive and natural way.

Quetzal and Term [32] are tangible languages used for teaching programming. They consist of blocks that can be connected with each other to represent programming structures, actions and parameters. In this way students construct their own programs easily and are able to identify errors in the code faster. Programming with tangible interfaces also facilitates collaborative work. Other examples of tangible programming languages are Turtan [33] and TanPro-Kit [34].

Tangible interfaces have also been used within computer education for teaching databases. Ullmer *et al.* [35] propose a tangible system that enables the creation, manipulation and visualization of database queries. Another example in this area is CubeQuery [36].

There are also applications with tangible interfaces for teaching mathematics [37], teaching music [38] and teaching language [39].

As in the case of virtual worlds, tangible interfaces provide benefits for inclusive education. For example, Muro *et al.* [40] propose the use of tangible interfaces with children with Down syndrome. In their system students relate tangible objects with words or sounds, under the Troncoso and Del Cerro method.

Jadan-Guerrero *et al.* [41] also propose using tangible interfaces for children with intellectual disabilities using the Troncoso and Del Cerro method. The usefulness of the system in the early stages of literacy is proven, and it is also shown that tangible interfaces facilitate thinking through physical actions. In both cases an initial increase in the interest and attention of students is demonstrated, but it has to be further studied whether it is maintained in the long term.

Topobo [42] is a building system with kinetic memory aimed to autistic children. They have found improvements in collaborative and social skills, including the reduction of the risk of loneliness.

50% of the projects mentioned above use as tangibles pieces that fit with each other (e.g., pieces of wood as in the classic jigsaw). The other 50% of projects use tangible elements representing real objects (for example, to associate a word to an object); tangible elements that can be assembled in pieces to create a shape, person or animal (for example Topobo, which allows creating a dog or a dinosaur); or special elements (for example, a magic carpets for capturing positions). In all cases very specific tangible elements have been developed to carry out the projects. This makes difficult for teachers to have their own kit of tangible elements to develop their own projects. In this paper, we propose defining a standard set of tangible elements, with a standardized communication mechanism, which teachers may use themselves for the creation of educational activities.

### 1.3. Mixed Reality

In this paper, we propose using virtual worlds and tangible interfaces to create a “mixed reality” experience. The term “mixed reality” can be defined as the space where the real and virtual worlds merge [43]. There are several ways of creating mixed reality applications.

For example, it is possible to create meeting spaces that are half physical and half virtual. MiRTLE (“A Mixed Reality Teaching and Learning Environment”) [44] goes in that direction: students in the classroom and remote students interact with the teacher and with each other using simultaneously the physical and virtual worlds. 

SMALLable [45] is a framework for mixed reality systems that allows teachers and students to work collaboratively designing different scenarios. For example, a scenario was developed in which displays and auditory cues were used to represent chemical information.

The MARVEL project (Virtual Laboratory in Mechatronics: Access to Remote and Virtual E-learning) [46] is a European project that aims to implement and evaluate learning environments for mechatronics in vocational education. Students are allowed to access real laboratories through virtual worlds, thus providing environments that merge the real and virtual under the paradigm of mixed reality.

TIWE Linguistico [47] is a competition that allows high school students from Italy learning English using a mixed reality environment that includes Android mobile devices and virtual worlds. In this competition each team has a virtual group that has to find out in a virtual world who has perpetrated a crime (based on the novel “The Hound of the Baskervilles”), and a real group that, using mobile devices and QR codes, will answer a series of questions about it. 

Perhaps one of the most appropriate fields for the application of mixed reality is geometry. Nikolakis *et al.* [48] proposes a mixed reality system that has been tested in secondary schools. This system uses a virtual environment for creating 3D objects and a haptic glove that allows interaction with the virtual geometric objects. The results obtained through several questionnaires indicate that it is an efficient way to solve geometry problems.

Finally, Magic Book [49] is an example of a tangible book that allows looking at pictures or reading texts in a traditional way and also, using special glasses (a head mounted display), to watch animated models and 3D virtual scenarios on top of the pages. ARToolkit was used to create the position marks that are added to the pages for the later incorporation of the animated virtual scenes.

As seen in the mixed reality projects reviewed above, a fairly significant infrastructure is needed: special glasses for viewing three-dimensional elements in the scene, special haptic gloves, *etc.* Specific visualization software is also needed. The reviewed projects are devised as prototypes for “proof of the concept” experiences, most of them not having a vocation for sustainability. 

Our proposal puts emphasis in this “sustainability” aspect. The idea is to provide teachers with a toolkit that will allow them to develop their own educational applications.

## 2. Virtual Touch: A Toolkit for Developing Mixed Reality Educational Applications

In this paper, we present Virtual Touch, a toolkit that allows the development of educational activities through a mixed reality environment where, through various tangible elements, we can interconnect a virtual world to the real world. The main goal of Virtual Touch is to facilitate the installation, configuration and programming of different types of technologies, abstracting the creator of educational applications from all technical details involving the use of tangible interfaces and virtual worlds. Therefore, it is specially designed to enable teachers to create educational activities for their students in a simple way, considering that teachers generally lack of advanced knowledge in computer programming and electronics. 

In this section, we will describe the architecture that has been developed to allow the development of educational applications based on mixed reality and the toolkit elements that have been created using this architecture.

### 2.1. Virtual Touch Architecture

Virtual Touch aims to be a system easy to use and configure, available to users without special knowledge of computers, but also flexible enough to be useful for users with more advanced knowledge in programming and electronics. The later may get involved in more advanced settings and even may have the possibility of adding new components to the system. Facing such flexibility and adaptability we have distinguished three types of users that can use the toolkit for the development of educational applications:

- **Basic user:** is a teacher who has a basic knowledge of computers and only have to know how to launch the educational programs provided to her; how to connect to the system the tangible elements needed for the activity (among those available within the toolkit); and how to perform a basic configuration of some parameters of the educational application.
- **Intermediate user:** is a teacher who has some knowledge of computers, being able (for example) to create macros in spreadsheets, to create objects and to edit scripts in virtual worlds, or to manipulate hardware elements in toolkits like Lego Mindstorms. This user may use Virtual Touch to create new educational applications, building her own virtual world and connecting it to the real world using the tangible elements available in the toolkit.
- **Advanced user:** is a teacher with extensive knowledge in programming and electronics, who will be able to add new modules to the system. This kind of user may add new elements (for example, creating new tangible elements using sensors and actuators that are not present in the initial toolkit) or may use different hardware technologies from the three contemplated in the toolkit as supplied.

To meet the needs of these three types of users the Virtual Touch architecture uses the following elements:

• A virtual world server.
• A virtual world client.
• A set of hardware elements: the tangible interaction devices.

The proposed architecture connects these elements through a middleware that coordinates tangible interfaces with the virtual world. The middleware includes a part that interfaces with the virtual world and a part that interfaces with the tangible elements.

The initial version of Virtual Touch includes three specific technologies for creating tangible elements, but it is designed so that, if necessary, new hardware technologies can be incorporated in a modular way. The hardware technologies initially contemplated are Phidgets, Arduino and Microsoft Kinect.

We used the Phidgets technology taking into account that it is easy to use, it has an affordable price and it provides many useful sensors. Arduino technology has also been integrated in our architecture, being a technology widely used worldwide, reasonably affordable and which also provides many useful open hardware sensors. Both Phidgets and Arduino technologies allow the integration of countless sensors that react to properties such as humidity, temperature, light, force, motion, acceleration, *etc*. For a developer, Phidgets technology stands out for its ease of use, while Arduino is more flexible. Finally, the Microsoft Kinect technology has also been integrated, as a complementary means for including gesture recognition, voice recognition, and shape recognition (using additional libraries such as OpenCV).

With respect to the technology for constructing virtual worlds, OpenSim has been chosen. This technology, on one hand, is compatible with one of the most extended virtual worlds, Second Life, and on the other hand it is open (and free) software. This facilitates that teachers learn easily how to create, deploy and maintain their own virtual worlds. There are many materials and help pages available in the Web for learning how to create virtual worlds using such technology. In fact, there are already some teachers already trained and using Second Life for their teaching. It also facilitates that the expenditure required by schools is not very high and that the work needed for the integration of the tangible interfaces is feasible.

**Figure 1:**
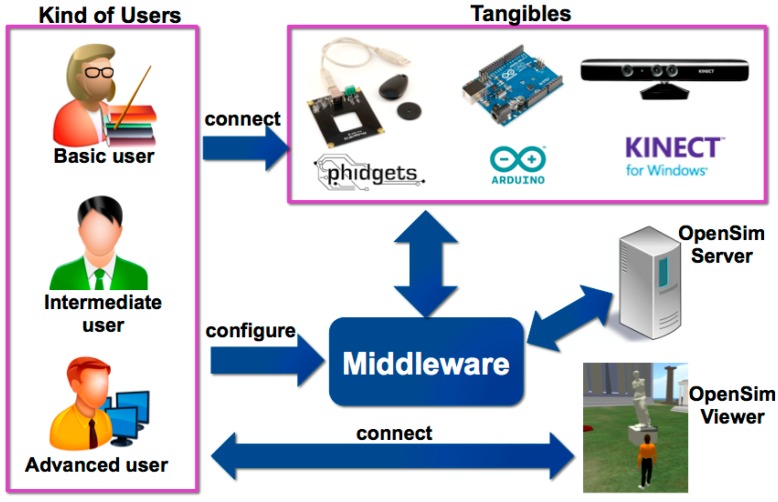
Elements that interact in the architecture.

In short, the architecture provides for three types of users three different technologies for creating tangible interfaces and a technology for creating virtual worlds, as shown in Figure 1.

**Figure 2:**
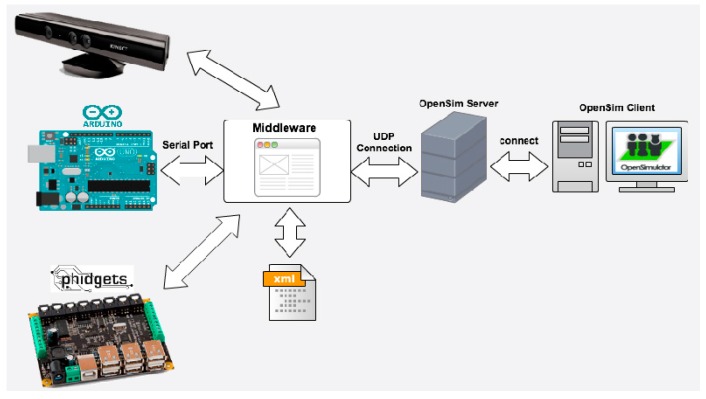
Virtual touch hardware architecture overview.

Once the various actors in our system were identified, the next step was to design the hardware architecture. The core of the architecture is a middleware that has specific modules to interact with each of the selected hardware technologies (see Figure 2). The middleware processes the events that happen in the tangible interfaces (which are built with one or more of these three technologies), and communicates them to the OpenSim server. The student perceives the outcome of the interaction through the OpenSim client (a viewer). The interaction of the tangible interfaces with the virtual world can be configured via a configuration file. Normally both the middleware (and therefore the tangible interfaces) and the OpenSim client will be housed in the student’s computer, while the OpenSim server is hosted on a remote computer. This, however, is flexible in the proposed architecture, allowing other settings if necessary. For example, for efficiency reasons we may host the middleware and tangible interfaces in a third computer, or conversely for simplicity reasons we may host all the software in a single computer (the student computer). In the latter case the virtual world would behave more like a video game, with no possibility of interaction with other students.

**Figure 3:**
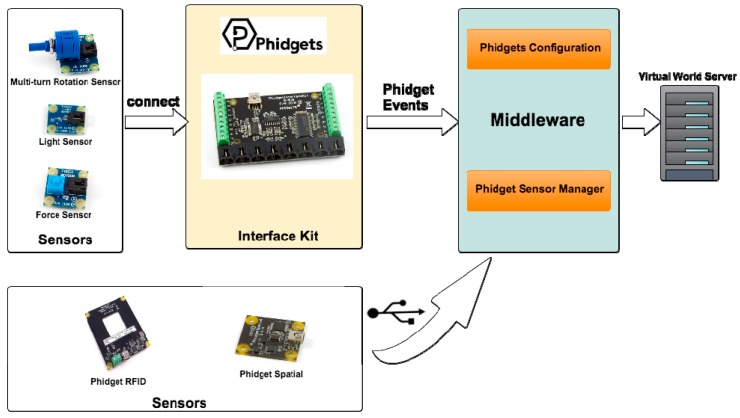
Hardware architecture for the Phidgets technology.

In order to specify in more detail the architecture, we have to describe separately each of the three selected hardware technologies. For example, the detail of the hardware architecture for the “Phidgets” technology is shown in Figure 3. Some elements of this technology are connected through an “Interface Kit” while other elements are connected directly to a USB port. The middleware manages the different sensors regardless of whether they connect via Interface Kit or not. The sensors that are connected via the Interface Kit will be automatically configured (by the middleware), specifying at which port on the Interface Kit each sensor or actuator is connected.

**Figure 4:**
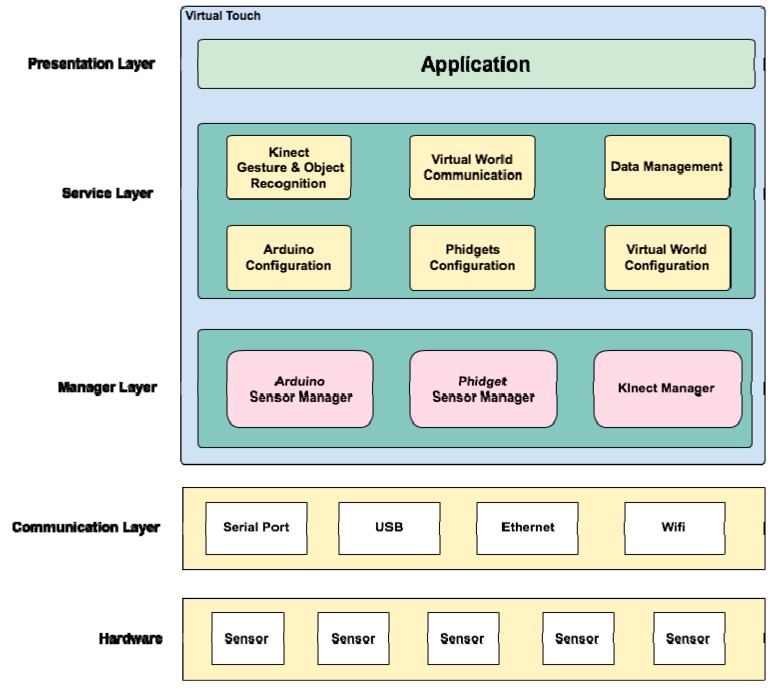
Virtual Touch Software Architecture.

On top of this hardware architecture we designed a multi-layer software architecture that consists of the following layers (see Figure 4):

- Communication layer: this layer is the closest to the hardware, and therefore to the physical devices. It defines and manages the kind of connection that will be used, such as USB, WiFi, Ethernet, Bluetooth, *etc.*
- Device management layer: this layer enables the interaction of the middleware with the three selected hardware technologies: Kinect, Arduino and Phidgets. This layer arranges for proper communication among the middleware and the sensors/actuators that conform the tangible interfaces.
- Service layer: the following services are available in this layer,
  ○ Gesture and Object Recognition: this service is responsible for gesture recognition, voice recognition and forms recognition (it uses third part libraries such as OpenCV).
  ○ Virtual World Communication: This service is responsible for sending and receiving messages to and from the OpenSim server. This service manages the data captured by the different sensors and actuators connected to Virtual Touch system.
  ○ Data Management: This service is responsible for data management and storage. In particular, it stores some activities and parameters in a MySQL database. For example, multiple choice questions and answers (for tests) may be stored here, using a persistence mechanism.
  ○ Configuration Services: This service is responsible for the configuration of the mixed reality system (for example, for specifying which OpenSim server, local or remote, is used). In the case of elements using the Arduino or the Phidgets technology, these services will allow the configuration of the ports where the various sensors and actuators are connected.
- Presentation layer: this is the layer closest to the user, in charge of displaying the graphical information. It is comprised mainly by the virtual world engine, and allows the configuration of how the interaction with the tangible interfaces is shown in the virtual world.

### 2.2. Virtual Touch Toolkit

As explained above, Virtual Touch can be used by three types of users (teachers): basic users, intermediate users and advanced users. The difference among them is related with their knowledge about computers.

The basic user corresponds to a teacher whose knowledge of computers is elementary. She requires that the system is easily set up, its configuration being as basic as possible. This type of teacher may use educational applications already created (and provided with the toolkit), perhaps after setting some parameters, using the tangible interfaces that are also provided as part of the Toolkit.

The basic user will most likely not be able to perform the interconnection of the different sensors and actuators, lacking of the necessary knowledge in electronics. For this reason, the Virtual Touch Toolkit provides tangible components with already prepared connections, so that the basic user (and even the intermediate user) does not have to know these low-level details. Nor she has to worry about programming tasks (e.g., programming the Arduino microcontroller), as the middleware includes a “standard” configuration that allows the connection of the available tangible modules at predefined ports.

For example, for the Arduino technology the middleware predefines (for the basic user) the A0 analog pin for the force sensor, the analog pin A1 for the infrared sensor, the A2 analog pin for the light sensor, and so on for the rest of the pins, both analog and digital. Besides, the needed wiring has been encapsulated in black boxes.

The intermediate user is a teacher whose knowledge of computers and programming is enough, for example, to program macros in spreadsheets, or to create objects and write scripts in virtual worlds. She is not a computer specialist, nor has extensive knowledge of programming, but she is able to perform some relatively advanced tasks in these fields. This type of user can work with the applications and tangible interfaces provided with the toolkit (such as the basic user does), but she also can create her own educational applications of virtual worlds, either by modifying existing ones, or by creating them from scratch, using the provided tangible interfaces. In some cases she may even develop a new (very basic) tangible device, using the sensors and actuators already available in the Toolkit.

The advanced user, which may also be the teacher but most likely will be an auxiliary person who will help the teacher making her educational application, is an expert in programming. She can use the Toolkit in the basic or intermediate mode, as described above, but she is also able to create new tangible interfaces using sensors and actuators that are not covered in the current version of the toolkit (adding functionality to the middleware). She may even be able of adding new hardware technologies to the three currently supplied (adding new modules to interface with the middleware).

**Figure 5:**
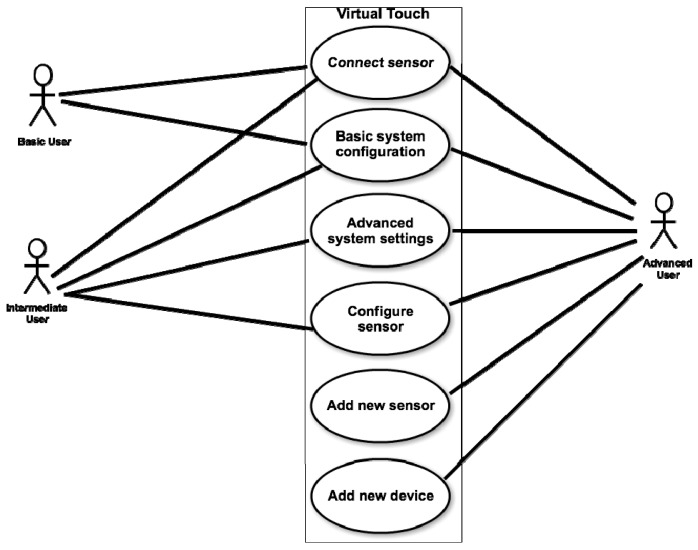
Virtual Touch diagram of use cases.

Figure 5 shows a diagram of use cases that shows the three types of users of the toolkit and the actions that they can perform.

**Figure 6:**
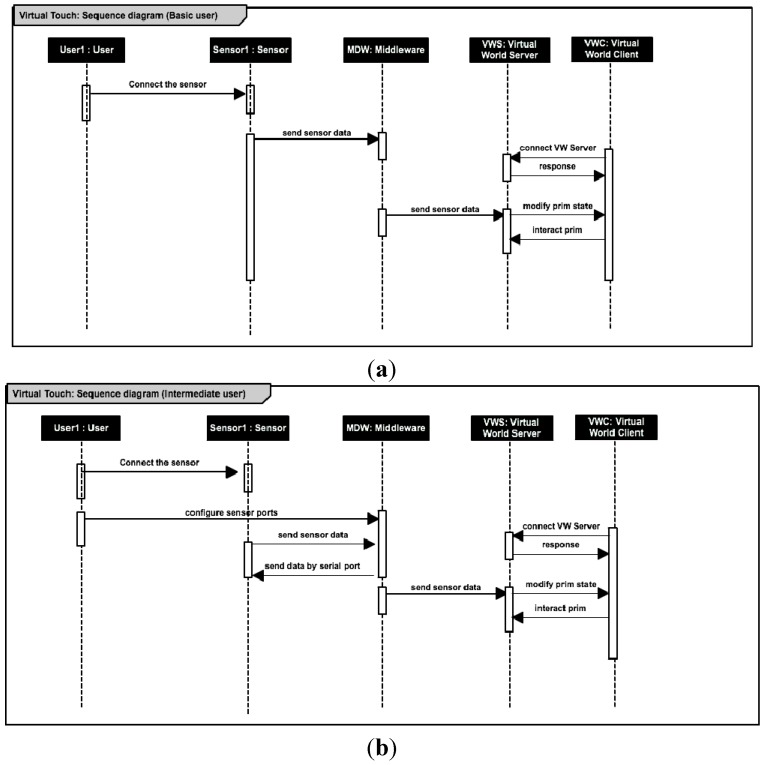

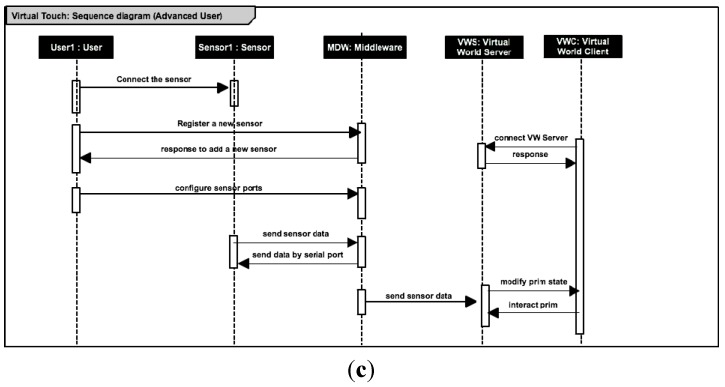
Sequence diagrams of the actions that the basic user (**a**); the intermediate user (**b**) and the advanced user; (**c**) can perform in order to interact with the sensors.

The actions that the different kinds of users can perform to create educational applications with the Virtual Touch toolkit can be formalized in the following sequence diagrams (Figure 6).

The VirtualTouch toolkit provides a library and an associated middleware for connecting the input from the different sensors with the virtual world. For example, in the case of the Kinect sensing device the middleware in the first place is responsible for initializing it (initializes the color, depth, pixels, *etc.*) The middleware then waits for the detection of a “blob” (a new object in the area of vision), by means of an event handler activated by the Kinect device. When such event occurs, the middleware transforms he image of the camera (originally in color) to black and white and eliminates noise. Then it uses this information and the information of the “depth sensor” (infrared) to recognize the shape of the “blob”. The OpenCV library is used for this task. Having identified the geometric figure, the middleware sends a message to the virtual world indicating the geometric figure and its position (relative to other geometrical figures at sight). Inside the virtual world a corresponding part of the middleware receives the message and makes the information available to any script in the virtual world that may need it. All of this is absolutely transparent to the teacher that is developing a virtual world educational application. She will construct the world in the usual way, and will find the information about the tangible blocks available when she needs it.

## 3. Case Studies

The architecture and toolkit presented in this article have been developed with a spiral methodology. After taking the initial requirements, a preliminary design was done and a first version of the architecture, in the form of middleware and associated tangible elements, was implemented. This was used for a prototype of a mixed reality educational application, which finally was employed by real students. From what was learned in this experience additional requirements were extracted, a refined design was done, and a new version of the architecture and a new educational application were implemented, repeating the cycle three times, as shown in Table 2.

**Table 2:** Case studies.

Architecture and Toolkit	Name	Tangibles and Devices	Virtual World Engine	Educational Application	Reference
Virtual Touch	Cubica	RFID reader and cubes with RFID tags inside. Wooden box simulating a vector.	OpenSimulator	Learning sorting algorithms for high school students	[50]
	Virtual Touch Eye	Kinect device, wooden figures (square, triangle, pentagon)		Catalan language learning for pupils with special educational needs	[51]
	Virtual Touch Book	Cardboard book with light sensors using Arduino.		Learning Catalan Language and learning Classical Greece culture	[52]

The three study cases follow a social constructivist approach through collaborative and cooperative learning. In each of them there are different kind of activities (student-centered activities) with the following characteristics:

- Encourage the active participation of students.
- Adapted to different levels.
- Encourage interaction between students and teacher.
- Allow the development of simulated training spaces
- Provide immediate feedback.

In this section, we present in more detail these three educational applications, which have been developed so far with the architecture and toolkit presented in this paper, as well as the related experiences performed in two secondary schools.

### 3.1. Cubica

The main learning objective of Cubica is to facilitate teaching sorting algorithms (bubble sort, selection sort, and insertion sort) in a more enjoyable and pedagogical way. This is done by means of a physical representation of arrays in the form of a wooden tangible. The tangible that represents the array facilitates the tracking of each iteration of the algorithm, reducing the complexity that is associated with abstract concepts. Manipulating the objects being sorted makes the problem much easier to understand. When the student changes the position of one of the cubes (representing numbers) in the tangible, the virtual world array also changes accordingly, and feedback is provided about the correctness of the move.

The idea is that the process of “ordering” is more familiar to students if done physically. The Cubica tangible interface facilitates the monitoring of each of the iterations that are performed while applying a particular sorting algorithm. The student physically manipulates cubes representing numbers, changing their position within a row. The actions in the real world with the tangible interface have immediate effect in the virtual world, where the activity is taking place. The student receives feedback in this way. The array is implemented by a tangible wooden model that has positions where the cubes can be placed. Each cube position is detected by a mechanism based on RFID sensors. There is also an LCD screen that reports the iteration that is taking place while performing the sorting algorithm. 

The base technology used for the tangible elements has been “Phidgets” [53], which is a simple and relatively inexpensive technology that allows the use of many different sensors. In the case of Cubica we have used RFID tags and readers, as well as an LCD screen.

**Figure 7:**
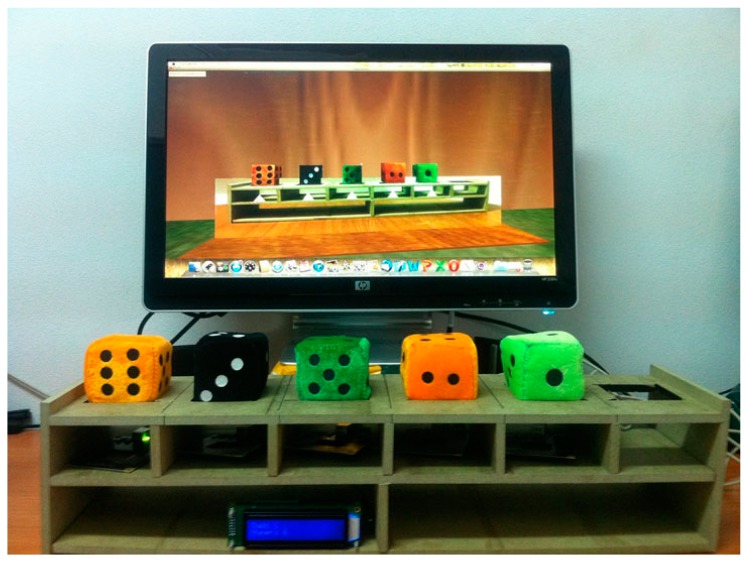
The Cubica educational application.

Figure 7 shows the Cubica application. On the screen, the representation of the array in the virtual world “Algorithms Island” is displayed. In this island students can perform different educational activities related to sorting algorithms. Any action in the tangible interface has an immediate effect in the virtual world, first making the virtual array to match the tangible interface, and then triggering the adequate events in the virtual world, depending on whether the movement realized by the student was correct or not.

Various educational experiences using the Cubica application were conducted in a public secondary school in Castellón (Spain): the IES Joan Coromines. 

The first session consisted of learning to use the virtual world: changing the look of the avatar, exploring the “Algorithms Island”, creating objects and programming them (using the language LSL), *i.e*., a “classic” use of a virtual world in education.

**Figure 8:**
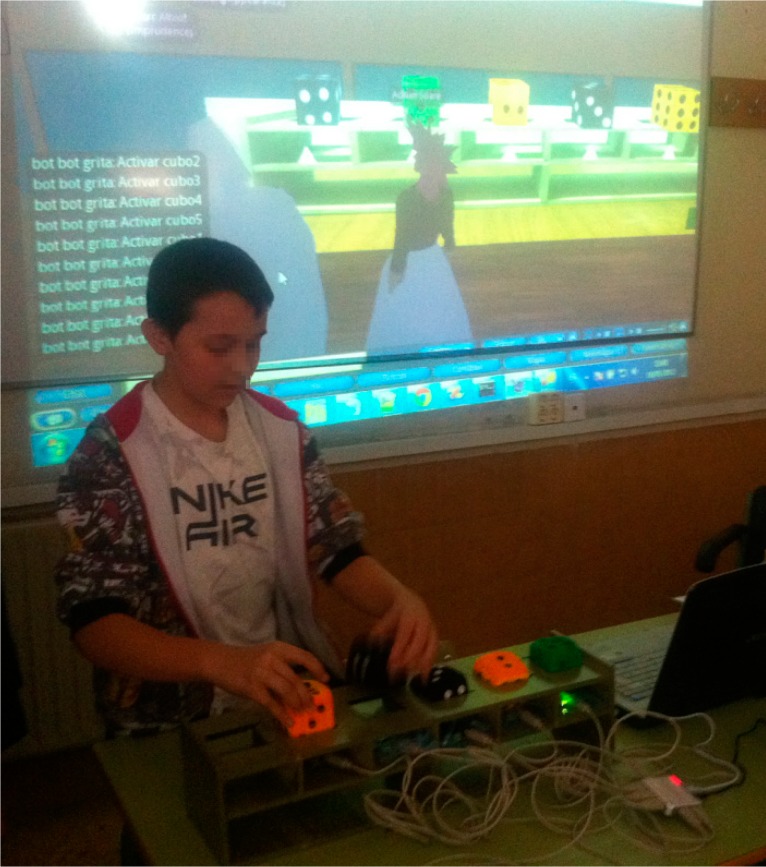
A student using Cubica.

In the second session the concepts of algorithms, array, loops and iterations were explained as well as the tangible elements provided by Cubica to practice with these concepts. The students explored the “Algorithms Island”, finding and experimenting with the different “themed houses”, which were designed to explain each type of sorting algorithm (see Figure 8).

In the third session, the students continued practicing with different exercises using Cubica. The performance was finally evaluated by means of tests on the subject that the students took and questionnaires for evaluating the perceived usability and usefulness of the system both by students and the teacher.

As was commented above, questionnaires were used to assess the perceived impact of system. Cubica was perceived as very helpful for understanding the operation of sorting algorithms (97%). The students thought that the system allowed them to better understand the concept of array (95%). Cubica helped distinguishing the different sorting algorithms from each other (88%), and it seemed interesting and motivating to all the students. 83% of them said they actually would like to continue working with the system at home. The worst factor in the experience was the distraction aspect that is involved when using virtual worlds: some students spent too much time customizing their avatar and exploring all parts of the island, without adhering properly to the proposed timing and objectives. More details on this educational application and its results can be found in [50].

Cubica was the first application created with the Virtual Touch architecture and toolkit, being also the testbed in which the main ideas of this architecture were developed. The results of this experiment were:

- A first version of the Virtual Touch middleware, which allows the easy creation of mixed reality applications.
- A concrete tangible interface, consisting of a series of cubes that can be physically manipulated, and whose manipulation has an immediate effect in the virtual world. This tangible interface becomes part of the toolkit available for future applications.
- A specific educational application, including a virtual world (the “Algorithms Island”), with a set of activities, many of which make use of the tangible interface. This application also becomes part of the toolkit available to third parties, either for using it directly “as it is” or for modifying it to create other educational applications.
- A case study of the above in an educational secondary school.

### 3.2. Virtual Touch Eye

In the second iteration of the development cycle of the Virtual Touch architecture, the educational application VirtualTouch Eye was developed. This application used wooden figures as tangible elements, representing shapes (a cube, a pentagon, a hexagon, *etc.*) that were recognized by a Kinect device [54]. 

The integration of a Kinect device in the Virtual Touch architecture allows various new types of activities. It adds to the toolkit the possibilities of gesture recognition, recognition of basic shapes and even speech recognition, all of them quite useful for creating tangible interactions.

**Figure 9:**
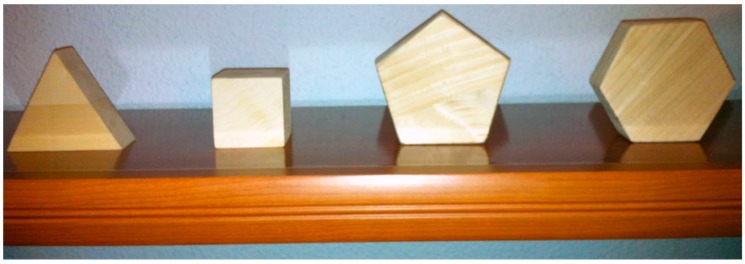
Some tangible objects used in Virtual Touch Eye.

The Virtual Touch Eye application employed for the interaction a set of tangible objects (wooden figures representing cubes, pentagons, hexagons, *etc.*) that were manipulated by the students (see Figure 9). The manipulations had immediate effect in the virtual world.

The learning objective of the Virtual Touch Eye case study is learning the grammar structures in the Catalan language. To do this, students learn the structure and the parts of a sentence by interacting with tangible elements that represent the different elements of a sentence. Physical interaction allows a “playing” and manipulative approach to such an abstract subject as grammar. Wooden blocks represent the different parts of the sentence, and finding the right grammar structure is transformed in a task that is more concrete for the student.

This educational application was created and tested at the IES Ernest Lluch, a high school in Tarragona, Spain. In this school the rate of immigrant students is high and they have a problem with the newcomers, which need to learn the Catalan language in order to be incorporated into regular classrooms. To this end, a number of courses are offered by the Catalan educational system, which are called “Welcome Courses”. These courses allow immigrant students (between 12 and 15 year old) to receive an additional and specific training in Catalan language and culture, as well as other transversal skills.

In this experience the tangible wooden figures were used to physically represent abstract concepts. For example, cubes represented names (in a sentence), triangles represented verbs, and spheres represented adjectives. Different types of activities for practicing creating sentences or identifying the main elements of a sentence were developed in the virtual world using these tangibles.

**Figure 10:**
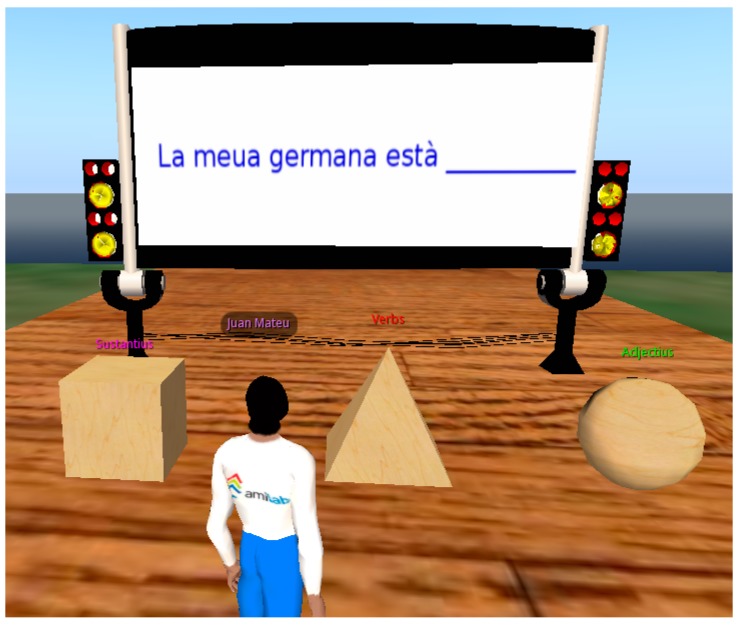
Example of exercise using the tangible elements.

Figure 10 shows an example in which the student had to guess what kind of word is missing in the blank space (a noun, a verb or an adjective). The student showed the tangible element corresponding to the type of the missing word.

Another type of activity was to identify the subject and predicate of a sentence. To do this, the sentences were divided into two parts, and the student placed the part he believed was the subject is in its place, followed by the part he identified as the predicate. All the activities in the virtual world produced feedback after completion, indicating whether the activity was completed successfully or not. 

The system was used in four sessions in 2 days, involving students from several regions of Spain and from several other countries: China, Ukraine, England, Colombia, Cuba, and Morocco. The students came from various course levels and had various levels of background knowledge of Catalan language and culture. The activities were performed by the students, and the teacher assessed the grade of success of the activity. According to these assessments (obtained from semi structured interviews with the teacher after each experiment), the students found the system easy to interact, both using gestures and tangible artifacts, and the tangible elements in particular helped the understanding of the different parts of speech, especially in cases of students whose language is quite different from Catalan. The complete table of findings from this experiment can be found in [51].

The results of this educational experience provided the Virtual Touch architecture and toolkit the following:

- A second version of the Virtual Touch middleware that, in addition to correct and optimize different aspects of the interaction with virtual worlds, added the possibility of incorporating tangible interfaces based on image recognition.
- A set of libraries and tools that enable the development of tangible interfaces based on image recognition. Thus, in this version of the middleware, two possible technologies for the development of tangible interfaces were contemplated: Phidgets and Kinect.
- A set of tangible elements in the form of geometric figures, so that the physical handling of the figures has immediate effects in the virtual world. This set of tangible items (and the libraries that incorporate their handling into the virtual world) are available on the Virtual Touch toolkit, and can be used for the development of future educational mixed reality applications.
- An example of educational application using mixed reality that is available within the Virtual Touch toolkit for possible employment “as it is” or for adapting it to new (similar) applications.
- A case study of the use of mixed reality systems in real environments.

### 3.3. Virtual Touch Book

In the third iteration of the development cycle of the Virtual Touch architecture and toolkit, the Virtual Touch Book was developed. It is a mixed reality book that allows reading a tangible book in the traditional way, but complementing the reading with activities in a virtual world. According to the book page being read, a series of activities are activated in the virtual world. The Virtual Touch Book allows reading the theoretical contents in a tangible book and then going to the virtual world to put into practice these theoretical contents.

The Virtual Touch architecture captures the page being read in the book and sends that information to the virtual world. Arduino technology [55] is used to detect the page being read, by means of LDR sensors that measure the amount of light on each page. The page most illuminated is the current page. Thus, Arduino technology has been added to the Phidget and Kinect technologies that were previously available for the development of tangible interfaces.

The Virtual Touch Book is an example of situated learning. Students are immersed in a specific context, the Ancient Greece, with the objective of learning the Greek culture. We use non-player characters (NPCs) in order to give information to the students. This mechanism allows students to build their knowledge using constructivist principles through interaction and socialization with the avatars present in the virtual world, enriching their learning process by providing a realistic context. According Dede [56], the NPCs provide intellectual and psychosocial feedback to students by means of interactions that are similar to those occurring in face-to-face constructivist learning.

The learning objective behind Virtual Touch Book is to enrich the experience of reading a paper book with a virtual world. There are several ways of doing this. For example, the virtual world may show additional materials (in a 3D realistic environment) to what is said in the book. These materials can be games (allowing game-based learning); examples to be solved by the student (allowing case-based learning); *etc*.

In the case study presented in this paper the virtual world is used to encourage the student to study carefully the book. Depending on which page of the book is currently studying the student, the virtual world proposes a game that can be solved only if the student understands what she is reading. In this way, the student is encouraged to work on the book to obtain good results in the game. Game-based learning is pedagogically efficient because it encourages students to be more active and committed in the activity.

The Virtual Touch Book has been tested in the IES Ernest Lluch, a high school in Tarragona, Spain. For this we created a virtual world representing the Ancient Greece, importing three-dimensional models of monuments, buildings and classic statues form public libraries available on the Web, and creating a series of activities, all of them related with the materials contained in the book.

The experience was performed with various groups of pupils with special educational needs. The goal was to teach various aspects of the Classical Greece, within the “Religion and Greek mythology” section of the subject of Social Sciences. In this experience the students learned different aspects of the Classical Greece, such as the dressing the Greeks used, or the different Gods they worshiped.

**Figure 11:**
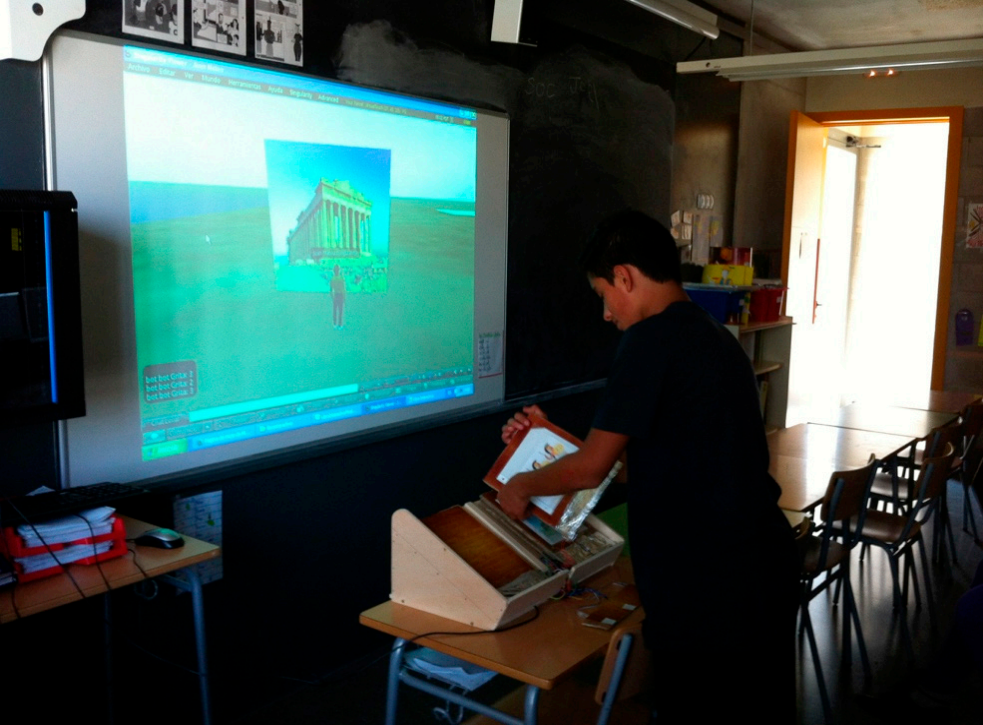
A student using the Virtual Touch Book.

In order to establish an experimental control, one of the student groups worked with the Greek gods using traditional methods, while the other groups worked with the Greek gods using the Virtual Touch Book. In each page of the book there were information and images about one specific Greek God. The students read that information to know about the god and its characteristics, and then went into the virtual world for a series of activities. The information obtained by the students reading the book served them to search for the god in the virtual world that simulates ancient Greece. After locating the god in question, the student obtained a token that had to be deposited in the Temple of Apollo. In case the god found corresponded with the god being studied in the book, the student had to answer some questions about it, getting a price if correctly answered. After receiving prices for three gods, the student obtained access to the Olympus (see Figure 11).

In this way students learn in a playful manner and immersed in a reward system (collecting prices, getting scores for correct answers, obtaining syllables to form a secret word, and finally accessing the Olympus), in an application of the concept of gamification [57].

At the end of the experience a test was delivered to compare the control group with the groups that used the Virtual Touch Book. In the control group (which used traditional teaching methods) the students achieved an average of 60% of correct answers in the test, while the groups using Virtual Touch Book achieved an average of 80% of correct answers. In general, it was found that using the Virtual Touch Book helped to hold the attention of students. More details on this educational application and its results can be found in [52].

The result of this educational experience provided the Virtual Touch architecture and toolkit the following:

- A third version of the Virtual Touch middleware that, besides correcting and optimizing different aspects of the interaction with virtual worlds, adds the possibility of incorporating tangible interfaces that are based on Arduino technology.
- A set of libraries and tools that enable the development of tangible interfaces based on Arduino technology. Thus, in the current version of the middleware three possible technologies for the development of tangible elements are contemplated: Arduino, Phidgets and Kinect.
- A tangible element in the form of a traditional book, which interacts with the virtual world so that the activities in the virtual world are triggered depending on which page of the book is being studied. The book is available as part of the Virtual Touch toolkit, and can be used for the development of future mixed reality educational applications.
- An example of educational application (the Ancient Greece Island) that uses mixed reality, which is available within the Virtual Touch toolkit for possible use “as it is” or to be adapted as a new (similar) application.
- A case study of the use of mixed reality systems in real environments.

## 4. Conclusions and Future Work

This paper has presented a toolkit and middleware that enable the creation of educational applications based on mixed reality. These applications can be created by teachers with different levels of experience in the use of computers: from teachers with no experience, to teachers with experience in programming and electronics. The first type of users use the toolkit “as it is”, using the applications, virtual worlds and tangible interfaces that are provided, perhaps changing some configuration parameters. Teachers with intermediate computer experience can create their own virtual worlds, making the interactions using the tangible interfaces that the toolkit provides. Teachers with more experience in programming and electronics can create their own tangible interfaces using the sensors and actuators that are provided by the proposed base technologies (Phidgets, Arduino, Kinect), or even they can add a new base technology, if they need to.

Three case studies have been performed, in which teachers with basic and intermediate levels of computer knowledge participated. The educational systems developed were tested with their own students, obtaining promising results. An experience is currently underway that involves users that have a more advanced level of computer knowledge, who are developing their own tangible interfaces and using them in an educational virtual world also developed by themselves. They are working from the examples that the toolkit provides. In the next year, a more comprehensive set of experiences with users of the three levels of knowledge will be held to confirm the usability of the toolkit and the educational efficiency of the applications developed. The resulting toolkit will be encapsulated and prepared for the possible distribution to third parties, including user manuals, software and hardware.

Comparing Virtual Touch with other mixed reality systems (see Section 1.3), the main contribution is the flexibility that Virtual Touch offers. The mixed reality systems that we have surveyed in the literature (see Section 1.3) do not involve full-fledged virtual worlds, but specific applications implementing concrete virtual environments (virtual reality). Although the difference is not important for a specific application, it is relevant when the objective is not a concrete educational application but a system that can be adapted for many different educational purposes. Thus, the main contribution of Virtual Touch is the possibility that offers to teachers to create their own mixed-reality educational applications, involving both virtual worlds and tangible interfaces.

Virtual Touch currently has some limitations that have to be solved in the future. Firstly, there is a concern with response times. The architecture has been designed to be flexible, but at the price of not being optimal from a response-time point of view. This has had some impact in the usability of the applications, depending on the speed of the communication networks. Each action of the user involves several messages among the modules of the middleware, which may be running in different computers far away from each other. During the experiments the virtual world server had to be installed in the same Local Area Network than the client computer in order to achieve acceptable response times. This has to be improved in the future. 

Secondly, the current implementation does not include any authoring tool to help the teacher to develop educational applications. Although the modular design of the system allows that development without the need of specific authoring tools, in a Lego-like style, however some tasks may be facilitated if they are provided in the toolkit.
